# Evaluating the Role of KRAS and NRAS 3' Untranslated Region Polymorphisms in Susceptibility and Clinical Features of Laryngeal Squamous Cell Carcinoma

**DOI:** 10.7759/cureus.76477

**Published:** 2024-12-27

**Authors:** Abdullah Albahar, Rana Sarmiti, Hamid Hossienzadeh, Massoud Houshmand

**Affiliations:** 1 Department of Surgery, Sheikh Jaber Al Ahmed Al Jaber Al Sabah Hospital, Kuwait City, KWT; 2 Genetics Department, Al Soor Clinic, Kuwait City, KWT; 3 Genetics, Pars Hospital, Rasht, IRN; 4 Medical Biotechnology, National Institute of Genetic Engineering and Biotechnology, Tehran, IRN

**Keywords:** cancer risk factors, kras, laryngeal squamous cell cancer, nras, single nucleotide polymorphism

## Abstract

Laryngeal squamous cell cancer (LSCC) is one of the most common head and neck cancers in which genetic factors play an important role in its occurrence. This study investigated the association of *KRAS *and *NRAS *gene polymorphisms with the risk of LSCC. *KRAS *polymorphisms including rs712, rs61764370, rs8720, and rs9266, as well as NRAS rs14804, were compared in the patient group (n=120) and the control group (n=100). The Sanger sequencing method was used to identify these polymorphisms. The results showed that *KRAS *rs8720 is associated with an increased risk of LSCC; consequently, those with the CT genotype were at a higher risk than those with the CC genotype. Also, the CC genotype had a protective effect on rs14804 polymorphism of the *NRAS *gene. These findings show that some *KRAS *and *NRAS *polymorphisms can be used as diagnostic and prognostic biomarkers in LSCC, and their accurate identification by Sanger sequencing is of great importance in research related to cancer genetics.

## Introduction

One prevalent cancer of the head and neck is laryngeal squamous cell carcinoma (LSCC) which has emerged as a public health issue due to its widespread occurrence and the considerable expenses involved in its treatment coupled with the risk of mortality associated with it [1]. LSCC is categorized into three types: LSCC located at the cords area of the voice box (glottis) supraglottic, LSCC above the vocal cords area, and subglottic LSCC, below the vocal cord region, each presenting distinct symptoms and prognosis [2,3]. This type of cancer makes up 2 percent of all cancer cases globally and is linked to a yearly death rate of 0.7 percent, among all cancer fatalities, particularly in individuals diagnosed late [4,5]. Research indicates that head and neck cancers like LSCC are forms of cancer, especially among men aged 50 and older who have higher exposure to risk factors, like smoking. The varying occurrence of this cancer type across countries may be attributed to factors like smoking patterns and cultural distinctions. Genetic traits and environmental elements such as smoking and alcohol intake stand out as the risk factors for LSCC. According to many studies that have been done, this type of cancer may be caused by some genetic mutations in a number of genes. Ras/Raf/MEK/ERK1/2 or mitogen‑activated protein kinase (MAPK) signaling pathway is responsible for regulating important biological functions such as cell survival, differentiation, growth, and migration [6,7]. Genes such as *KRAS, NRAS, *and* HRAS*, which are members of the RAS family, play an important role in this signaling pathway, and malignancies such as head and neck tumors are associated with the activation of these genes [8,9].

Single nucleotide polymorphisms (SNPs) in these genes have been found to be useful in controlling gene expression. By altering the binding locations of microRNAs in the 3′ untranslated region (3′ UTR), these SNPs disturb the homeostasis of the MAPK pathway and have an impact on the control of gene expression [10]. Mutations and polymorphisms of the *KRAS *and* NRAS* genes have been linked in certain studies to increased cancer aggressiveness, a greater recurrence rate, and a worse response to platinum treatments in patients with LSCC. *KRAS *mutations range from 4.3% to 11.5% of cases, whereas *NRAS *isoform mutations have been found in about 9.7% of LSCC patients [11]. These results imply that these polymorphisms can be utilized as biomarkers to forecast the severity of the disease and its response to therapy, in addition to their involvement in the occurrence of LSCC. Furthermore, several human malignancies, such as those of the breast, colon, and head and neck, have been linked to overactivation of the Ras/Raf/MEK/ERK1/2 pathway. By controlling a variety of proteins, this pathway is essential for cell growth and survival. Its elevated activity is linked to aggressive and recurring cancer phenotypes as well as a poor prognosis [12,13]. Thus, a thorough examination of *KRAS *and* NRAS* polymorphisms and how they affect the clinical manifestations of LSCC can aid in the identification of genetic variables that predispose people to the illness as well as the development of individualized and targeted therapies.

In this study, we investigate the relationship between the SNPs of the *KRAS* (rs712, rs61764370, rs8720, and rs9266) and *NRAS* (rs14804) and genes in people with LSCC and also the relationship between these SNPs and the clinicopathological characteristics of the patients and some risk factors which may play a role in the disease. The findings from this study could have applications in personalized medicine, including early diagnosis and prediction of disease progression. On the other hand, the association of these SNPs with disease improves understanding of the genetic role of these mutations in cancer development.

## Materials and methods

Study population and sample collection

This study included 120 patients with LSCC and 100 healthy individuals as a control group from Iranian men's society. The reason for selecting the male population for this study was the higher prevalence of this disease among this gender, increasing the homogeneity of the sample and preventing the impact of confounding factors such as gender differences in metabolism and lifestyle. The inclusion criteria of patients in this study are the age range between 45 and 75 years, confirmation of LSCC in patients based on pathology results, not having undergone previous chemotherapy and radiation therapy, and full satisfaction of participants. Also, exclusion criteria included patients with any type of cancer other than LSCC and having medical conditions such as kidney failure or uncontrolled diabetes. The control group consisted of healthy individuals with no history of cancer, matched for age and gender with the patient group. Each of the participant's age, gender, smoking status, and alcohol intake were among the clinical and demographic data gathered. This study was approved by the Ethics Committee of the National Institute of Genetic Engineering and Biotechnology (NIGEB Approval Number: IR. NIGEB.EC1397.11.39E). All procedures were conducted in accordance with the ethical standards outlined in the Declaration of Helsinki. Written informed consent was obtained from all participants prior to their inclusion in the study.

DNA extraction

From each participant in this research, 5 ml of peripheral blood was collected in EDTA tubes. Genomic DNA was extracted using a commercial DNA extraction kit (QIAGEN, Germany) according to the manufacturer's instructions. A spectrophotometer and 1% agarose gel were used to check the quantity and quality of the extracted DNA. 

Genotyping using the sequencing technique

To identify polymorphisms in* KRAS *and *NRAS* genes, Sanger sequencing technology was employed. Target regions containing known polymorphisms, including the 3'UTR, were amplified using specific primers (Table 1) and PCR. The accuracy of PCR was checked by 1% agarose gel electrophoresis; since the primers had to be specific, only one band for each product should be observed. Then PCR products were sequenced using the Genetic analyzer 3500 (Applied Bioscience, USA), and sequenced data were analyzed by chromas v.2.6.6 (Technelysium, Australia) and aligned by RefSeq data (KRAS: NG-007524.2 & NRAS: NG-007572.1).

**Table 1:** Primer sequences corresponding to each polymorphism in KRAS and NRAS genes

Gene	Polymorphism	Primer Sequence (5'→3')	Annealing Temperature (°C)	Product Length (bp)
KRAS	rs712	F: 5'- ATGACAGTGGAAGTTTTTTTTTCCTC-3' R: 5'- GAATCATCATCAGGAAGCCCAT-3	60	325
KRAS	rs61764370	F: 5’- CACTACCTAAGGACCGGGATT-3’ R: 5’- CCTGGTAACAGTAATACATTCCATTG-3’	57	467
KRAS	rs8720	F: 5’-TCT CCTTCTCAGGATTCCTACAG-3’ R: 5’-ACAAAGAAAGCCCTCCCCAGT-3’	59	131
KRAS	rs9266	F: 5’-CCAATTGTGAATGTTGGTG-3’ R: 5’-AATGTGAAAAGGAAATGG-3’	55	371
NRAS	rs14804	F: F: 5'- TGCAAATGTAGAGCTTTCTGG-3' R: 5'- CCTTTTTCCTAGAAGTGGTTTG-3'	57	397

Primer design

To investigate specific polymorphisms in *KRAS* and *NRAS *genes primer designed by primer 3 plus (https://www.bioinformatics.nl/cgi-bin/primer3plus/primer3plus.cgi), Table 1 provides the primer sequences and characteristics for each polymorphism.

Statistical analysis

Polymorphisms identified in *KRAS* and *NRAS* genes were analyzed in both patient and control groups. Chi-square tests and logistic regression were used to determine the odds ratio (OR) and 95% confidence interval (CI) to investigate the genotypic and allelic relationship of each of the SNPs. All analyses were performed using IBM SPSS Statistics for Windows, Version 26 (Released 2019; IBM Corp., Armonk, New York, United States) and the level of statistical significance was determined at p≤0.05.

## Results

Analysis of risk factors and pathological features in patients with LSCC

Table 2 compares the risk factors and pathological characteristics between a group of patients with LSCC (n=120) and a healthy control group (n=100). The average age of the patient group was 65.4 ± 6.7 years and that of the control group was 60.3 ± 10.2 years, which was not statistically significant. Regarding the smoking status, 71% of the affected patients were smokers, while this value was 32% in the control group, and this difference was reported to be statistically significant (OR: 5.16, 95 % CI: 2.90-9.17, p=0.00). In addition, 64% of affected patients had a history of drug use, while this number was 25% in the control group (OR: 5.37, 95 % CI: 2.98-9.65, p=0.00). Examining the pathological characteristics showed that most of the patients are in more advanced clinical stages (Table 2).

**Table 2:** Statistical relationship between risk factors and clinicopathological characteristics in LSCC patients and control group LSCC: Laryngeal squamous cell carcinoma The asterisk (*) in the "p-value" column indicates that values ≤ 0.05 are statistically significant.

Risk Factors and Clinicopathological Characteristics	Patient Group (n = 120)	Control Group (n = 100)	p-value*
Age (Mean ± SD)	65.4±6.7	60.3±10.2	0.18
Smoking Status			
Smokers	85 (71%)	32 (32%)	0.00*
Nonsmokers	35 (29%)	68 (68%)	
Alcohol Consumption			
Drinkers	15 (13%)	10 (10%)	0.56
Nondrinkers	105 (87%)	90 (90%)	
Drug Use			
User	77 (64%)	25 (25%)	0.00*
Nonuser	43 (36%)	75 (75%)	
Occupational Exposure to Chemicals	18 (15%)	12 (12%)	0.51
Family History of Head and Neck Cancer			
Positive History	13 (11%)	9 (9%)	0.65
Negative History	107 (89%)	91 (91%)	
Unhealthy Diet			
High-fat, Low-fiber Diet	76 (63%)	52 (52%)	0.09
Balanced Diet	44 (37%)	48 (48%)	
Disease Stage			-
Stage I	27 (23%)	-	
Stage II	36 (30%)	-	
Stage III	40 (33%)	-	
Stage IV	17 (15%)	-	
Lymph Node Involvement	48 (40%)	-	-
Metastasis	8 (7%)	-	-

Case-control analysis

The genotypic and allelic frequencies of candidate polymorphisms of the *KRAS* (rs712, rs61764370, rs8720, rs9266) and *NRAS* (rs14804) in the LSCC and control groups are shown in Table 3. Based on the results, some of these SNPs showed a significant relationship between the two groups, and some others were not statistically significant; and no association was found between them and LSCC. rs712, rs61764370, and rs9266 polymorphisms of the *KRAS *gene did not show any significant relationship with the risk of LSCC.

**Table 3:** Distribution of genotypes, alleles frequencies, and genotype models for polymorphisms at KRAS and NRAS The asterisk (*) in the "p-value" column indicates that values ≤ 0.05 are statistically significant.

Gene	Polymorphism	Genotype	Patient Group (n = 120)	Control Group (n = 100)	OR (95% CI)	p-value*	HWE
*KRAS*	rs712	GG	43 (36%)	35 (35%)	1	-	0.03
		GT	52 (43%)	41 (41%)	1.03 (0.56 - 1.89)	0.91	
		TT	25 (21%)	24 (24%)	0.84 (0.41 - 1.73)	0.65	
		Allele Frequency	G: 138 (58%) T: 102 (42%)	G: 111 (56%) T: 89 (44%)	1.01 (0.60 - 1.68) 0.93 (0.54 - 1.58)	0.96 0.79	
		GG vs. GT+TT	43 (36%) 77 (64%)	35 (35%) 65 (65%)	0.96 (0.55 - 1.67)	0.89	
		GG+GT vs. TT	95 (79%) 25 (21%)	76 (76%) 24 (24%)	0.83 (0.44 - 1.57)	0.57	
*KRAS*	rs61764370	TT	79 (66%)	68 (68%)	1	-	0.81
		TG	36 (30%)	29 (29%)	1.06 (0.59 - 1.92)	0.82	
		GG	5 (4%)	3 (3%)	1.43 (0.33 - 6.22)	0.62	
		Allele Frequency	T: 194 (81%) G: 46 (19%)	T: 165 (83%) G: 35 (17%)	1.01 (0.68 - 1.48) 1.13 (0.65 - 1.95)	0.95 0.65	
		TT vs. TG+GG	79 (66%) 41 (34%)	68 (68%) 32 (32%)	1.10 (0.62 - 1.93)	0.73	
		TT+TG vs. GG	115 (96%) 5 (4%)	97 (97%) 3 (3%)	1.40 (0.32 - 6.03)	0.64	
*KRAS*	rs8720	CC	64 (53%)	70 (70%)	1	-	0.67
		CT	49 (41%)	25 (25%)	2.14 (1.18 - 3.86)	0.01*	
		TT	7 (6%)	5 (5%)	1.53 (0.46 - 5.06)	0.48	
		Allele Frequency	C: 177 (74%) T: 63 (26%)	C: 165 (83%) T: 35 (17%)	1.17 (0.78 - 1.75) 1.96 (1.15 - 3.35)	0.43 0.01*	
		CC vs. CT+TT	64 (53%) 56 (47%)	70 (70%) 30 (30%)	2.04 (1.16 - 3.56)	0.01*	
		CC+CT vs. TT	113 (94%) 7 (6%)	95 (95%) 5 (5%)	1.17 (0.36 - 3.82)	0.78	
*KRAS*	rs9266	AA	37 (31%)	35 (35%)	1	-	0.28
		AG	55 (46%)	51 (51%)	1.02 (0.56 - 1.85)	0.94	
		GG	28(23%)	24(24%)	1.10 (0.54 - 2.25)	0.78	
		Allele Frequency	A: 129 (54%) G: 111 (46%)	121 (61%) 89 (39%)	1.00 (0.59 - 1.17) 1.70 (0.68 - 2.02)	0.97 0.54	
		AA vs. AG+GG	37 (31%) 83(69%)	35 (35%) 75 (75%)	1.04 (0.59 - 1.82)	0.87	
		AA+AG vs. GG	92 (77%) 28 (23%)	86 (86%) 24 (24%)	1.09 (0.58 - 2.02)	0.78	
*NRAS*	rs14804	CC	19 (16%)	41 (41%)	1	--	0.00
		CT	56 (47%)	30 (30%)	4.02 (1.99- 8.12)	0.00*	
		TT	45 (37%)	29 (29%)	3.34 (1.63- 6.85)	0.001*	
		Allele Frequency	C: 94 (39%) T: 146 (61%)	C: 112 (56%) T: 88 (44%)	1.81 (0.98- 4.29) 3.58 (1.95 - 6.55)	0.002* 0.00*	
		CC vs. CT+TT	19 (16%) 101 (84%)	41 (41%) 59 (59%)	3.69 (1.96- 6.94)	0.00*	
		CC+CT vs. TT	75 (63%) 45 (37%)	71 (71%) 29 (29%)	1.46 (0.83 - 2.59)	0.18	

*KRAS* rs8720 polymorphism was associated with disease risk, with the CT genotype having a higher risk than the CC genotype (OR=2.14, CI:1.18−3.86, p=0.01). In addition, the dominant homozygous model showed a significant relationship with increased disease risk (CC vs. CT+TT, OR=2.041, CI: 1.16 - 3.56, p=0.01).

Based on the results of the analysis and investigation of rs14804 of the *NRAS* gene, a significant relationship was observed regarding this SNP between the patient group and the control group. The analysis showed that the number of wild-type CC genotypes in the control group was much higher than in the LSCC group, Also, due to the significance of the dominant homozygous model between the two groups (CC vs. CT+TT, OR=3.69, CI: 1.96- 6.94, p=0.00), it can be concluded that the CC genotype probably has a protective effect and reduces the risk of LSCC.

Relationship between SNPs and clinicopathological characteristics of LSCC

The allelic model revealed an association between the minor allele T of NRAS rs14804 and advanced stages of cancer (OR 5.235; 95% CI: 1.492-15.709; p = 0.019). Additionally, patients carrying the NRAS rs14804 T allele showed a higher likelihood of positive lymph node status (OR 3.85; 95% CI: 2.15-8.91; p = 0.025). Also, the T allele of rs8720 of the *KRAS *gene showed a significant correlation with more advanced stages of LSCC (OR 2.34; 95% CI: 2.03-6.65; p = 0.04). Other analyzed polymorphisms of* KRAS* showed no associations with the clinicopathological factors of LSCC in either the genotype or allelic models.

## Discussion

According to the analysis of risk factors and SNPs in this study, it can be said that there is a complex interaction between environmental and genetic factors and the risk of LSCC and its precursor. Based on the findings, risk factors such as smoking and drug use were higher in the patient group than in the control group. The obtained OR shows that people with a history of smoking or drug use are more than five times more likely to develop LSCC. These findings are consistent with previous studies that point to the carcinogenic effects of tobacco and its combined effects with other risk factors. Hashibe et al. showed in their study that both alcohol consumption and smoking independently increase the risk of head and neck cancer, even in people who do not have one of these two risk factors [3]. On the other hand, in another study that investigated the relationship between opium addiction and laryngeal cancer, it was found that opium use acts as an independent risk factor for laryngeal cancer. The results indicate that the possibility of developing laryngeal cancer in opium users is about nine times higher than in nonusers [14].

However, genetic polymorphisms play an important role in increasing the risk of various diseases, including types of cancers, especially some SNPs of *KRAS *and* NRAS* genes, which are associated with a higher risk of LSCC. The rs8720 polymorphism of the *KRAS* gene, especially in individuals with the CT genotype, is associated with a higher risk of LSCC than the CC genotype, suggesting that alterations in *KRAS* may contribute to carcinogenesis by disrupting cell signaling pathways. Previous studies have provided results, not on LSCC, but on the role of KRAS polymorphisms, such as rs8720, and its role in increasing the risk of other cancers, including colorectal cancer. In one of these studies, the association of the CT genotype of SNP rs8720 in *KRAS* with an increased risk of colorectal cancer was proven. It was also found that the *KRAS* mRNA and protein levels increase significantly in people with this genotype [15]. Also, the rs14804 of the *NRAS* gene shows a protective effect for the CC genotype, while the presence of the T allele is associated with advanced disease stages and lymph node involvement, suggesting its possible role in tumor invasion and metastasis. The impact of SNPs in the 3'UTR region of the *KRAS, NRAS, *and *MAPK1* genes on the risk and clinical characteristics of LSCC was examined by Insodaite et al. This study, which was conducted on 327 men with LSCC and 333 healthy men, showed that the CC genotype in SNP rs14804 of the NRAS gene is associated with a reduced risk of LSCC. In addition, the T allele of this SNP has been observed in more advanced cases of the disease and positive status of lymph nodes [16]. SNPs in the 3'UTR region of NRAS and KRAS genes can play an important role in the progression of cancers including laryngeal squamous cell carcinoma by affecting gene expression and RNA regulatory interactions. The presence of microRNA (miRNA) binding sites in the 3'UTR region causes stability of mRNA and its translation. The occurrence of mutations such as SNPs in this area may change the way miRNA binds to mRNA and as a result increase or decrease the amount of protein expression [17]. Furthermore, the significant association of *KRAS *rs8720 and NRAS rs14804 polymorphisms with clinicopathological features of LSCC, such as advanced stages and lymph node involvement, highlights the potential of these SNPs as biomarkers to predict disease severity. Patients with these high-risk genotypes may benefit from more careful monitoring and more tailored treatment strategies. However, some *KRAS *SNPs in the present study, including rs712, rs61764370, and rs9266, did not show a significant association with LSCC risk, suggesting that the effect of genetic SNPs on LSCC may depend on other genetic or environmental factors. In a study by Insodaite et al., it was found that there was no link between SNPs rs712 and rs61764370 and their connection to LSCC; however, Jiang et al. on the other hand reported that mutations in the 3’ UTR of the *KRAS* gene can impact cancer risk through altering gene activation. Similarly, Huang et al. showed an important relationship between the *KRAS* rs712 T allele and metastasis, proposing that this could be a useful prognostic indicator in metastatic colorectal carcinoma patients [18,19]. Furthermore, in the research on examining the effect of the *KRAS* gene polymorphisms on breast cancer risk in Mexican women done by Gallegos-Arreola et al., the results for rs9266 were not conclusive and this association here was not accepted [20].

Limitations

The results of this study may be limited by factors such as limited sample size, focus on male gender alone, failure to examine environmental and genetic factors other than SNPs, and geographic and racial differences.

Overall, it is suggested that a larger-scale study is needed to confirm the role of* KRAS *and* NRAS *SNPs in association with LSCC. Furthermore, the integration of genetic screening in LSCC risk assessment could lead to improved early detection and improved patient outcomes. This study is in line with studies emphasizing the multifactorial nature of this cancer and also adds useful information to the growing body of studies investigating the combination of lifestyle and genetic factors in the pathogenesis of LSCC.

## Conclusions

According to the study's findings, some *KRAS *and *NRAS *gene SNPs can be associated with increased LSCC risk. In particular, the *KRAS* rs8720 and NRAS rs14804 SNPs play an important role in these patients, especially where certain genotypes are associated with a higher risk of disease progression and more aggressive clinical features, including lymph node spread and advanced cancer stages. These findings highlight the importance of genetic testing as one of the potential tools for predicting and early detection of cancer and better understanding the prognosis of this disease. Comparison with previous studies also shows that the role of *KRAS* and* NRAS *genes in other cancers is similar and may be effective in determining molecular pathways of cancer progression. In general, these results indicate the high value of these SNPs as potential biomarkers in the risk assessment and management of LSCC, and it is suggested that in future studies, more extensive research should be conducted to confirm these results and better understand the underlying mechanisms of these relationships.
